# Effects of Tonsil size on Pulmonary Function test Results after Tonsillectomy in Children 

**Published:** 2016-01

**Authors:** Mitra Samareh Fekri, Aliasghar Arabi Mianroodi, Hossein Shakeri, Narges Khanjani

**Affiliations:** 1 *Physiology Research Center, Kerman University of Medical Sciences, Kerman, Iran.*; 2 *Department of Otorhinolaryngology Head and Neck Surgery, Shafa Hospital, Kerman University of Medical Sciences, Kerman, Iran.*; 3 *Department of Epidemiology and Biostatistics, School of Public Health, Kerman University of Medical Sciences, Kerman, Iran.*

**Keywords:** Children, Spirometry, Tonsillectomy

## Abstract

**Introduction:**

Adenotonsillar hypertrophy is a typical cause of surgery in children. Evaluation and identification of patients as potential candidates tonsillectomy is a primary concern for otolaryngologists. This study focuses on the results of pulmonary function tests (PFTs) after tonsillectomy in children.

**Materials and Methods:**

This cross-sectional study examined 50 patients suffering from tonsillar hypertrophy in 2013. Full details and results of otolaryngology examinations were recorded. Moreover, patients were examined with respect to forced inspiratory flow at 50% of vital capacity (FIF50%), forced expiratory flow at 50% of vital capacity (FEF50%), forced expiratory volume in 1 second (FEV1)/peak expiratory flow rate (PEFR), and FEV1/forced expired volume in 0.5 seconds (FEV0.5) before and after surgery using spirometry. All data were analyzed using SPSS Software (version 19), and central descriptive measures, and data were compared by performing T-test and Chi-square tests.

**Results:**

According to tonsil size, patients were distributed as follows: 18 patients (36%) with +1 tonsil size, 18 patients (36%) with +2 tonsil size, and seven patients (14%) with +3 tonsil size, and seven patients (14%) with +4 tonsil size. Thirty-three (66%) and 17 patients (34%) were female and male, respectively, with a mean of age of 9.7±2.97 years (range, 7–18 years). Seventy-eight percent of patients were aged 10 years or less. Moreover, 25 patients (50%), 17 patients (34%), and eight patients (16%), respectively, reported obstructive symptoms, recurrent tonsillitis, and both symptoms. In patients with +3 and +4 tonsil size, spirometric parameters indicated relief of symptoms of obstruction. Only in patients with +4 tonsil size were the changes statistically significant.

**Conclusion:**

Tonsillectomy can relieve obstructive symptoms in patients with tonsils larger than +3 to a great extent. Additionally, spirometry can identify patients with +3 and +4 tonsils who do not have clinical signs of an obstructive upper airway.

## Introduction

Adenotonsillar hypertrophy, which is associated with different degrees of airway obstruction in children, is a common indication for adenotonsillectomy. In general, palatine tonsils and adenoids are small at birth, but increase in size from the age of 1 year to 4 years due to increasing immune activities (1). A number of different factors such as chronic bacterial infections and passive cigarette smoke, for example, cause adenotonsillar hypertrophy in children (2). Large tonsils are associated with symptoms of airway obstruction including mouth breathing, snoring, sleep apnea, coughing at night, and production of hyponasal sounds as well as sinusitis and recurrent otitis media (3). In severe cases, patients suffer from cor pulmonale, pulmonary artery hypertension, or reduction of alveolar ventilation; all of which may recur as a result of adenotonsillectomy (4). Severe obstruction of the upper airways causes cor pulmonale, disorders in ventilation, and reduction of alveolar ventilation (5), which in turn lead to symptoms of chronic hypercapnia and hypoxia and subsequently respiratory academia, pulmonary artery stenosis, right ventricular dilatation (6), and heart failure (7). Removal of airway obstruction by adenotonsillectomy reverses these conditions (8). 

The main characteristic of obstructive pulmonary disorders is a reduction in air flow, which can be measured by spirometry as a non-invasive technique (9). Pulmonary function tests (PFTs) may highlight the obstructive impact of adenotonsillar hypertrophy in cases where there is no clinical or radiological evidence of obstruction (10). Therefore, as a surgical indication, PFTs can be useful in children suffering from adenotonsillar hypertrophy (10). According to studies performed in Turkey, 27 out of 45 cases of mild obstructive pulmonary disease were resolved by means of surgery (11). In addition, all measured parameters such as forced expiratory flow at 75% of vital capacity (FEF75), mean forced expiratory flow at 50% of vital capacity (FEF50%), mean forced expiratory flow at 25% of vital capacity (FEF25%), mean forced expiratory volume in 1 second (FEV_1_), mean peak expiratory flow rate (PEFR), and mean forced vital capacity (FVC) were improved (12). Also, Yatav et al. (2002) demonstrated the respiratory abnormalities of patients during hypoxia; i.e. 50% reduction of FIF50% and 0.5% increase in FEF50%/FIF50%, FEV_1_/ forced expiratory volume in 0.5 second (FEV_0.5_), and FEV_1_/PEFR (all of which were significantly improved after surgery). Accordingly, spirometry can make a contribution to the diagnosis of adenotonsillar hypertrophy, prompting earlier decisions relating to surgery and helping to prevent cardio-pulmonary symptoms (13,14). Due to the high prevalence of adenotonsillar hypertrophy and its influence on the life quality of patients and even their appearance, and due to the importance of its early diagnosis to prevent other related symptoms as well the lack of detailed studies in Iran, the present study was conducted to investigate changes in PFTs in cases of adenotonsillar hypertrophy in relation to tonsil size before and after surgery.

## Materials and Methods

This cross-sectional study examined patients admitted to the Shafa Hospital (supported by the Medical Science University of Kerman) for tonsillectomy. Enrolled patients had a number of characteristics, as follows: tonsil size, +1 to +4; age range, 7–18 years; and informed consent provided by parents. Patients suffering from any disease which impacts upon pulmonary functions, including respiratory diseases and cardiopathy, upper airway obstruction such as nasal polyps, nasal septum deviation, and chest wall abnormality, were omitted from the study. 

All patients were visited by an otolaryngology resident who recorded their full details and results of an otolaryngological examination. Lower airway diseases were excluded by means of the patient history and physical examination. Also, the determined size of tonsils was to range between +1 and +4 on the basis of oropharyngeal obstruction: 25%(+1), 50%(+2), 75%(+3), and <75% (+4). All examinations were conducted by one person. All patients presenting with the required indications of tonsillectomy were examined through the following routine blood tests; hemoglobin (Hb), bleeding time (BT), clotting time (CT), international normalized ratio (INR), partial prothrombin time (PTT), and prothrombin time (PT) prior to surgery. Moreover, for spirometry conducted in Afzalipoor Hospital (Kerman, Iran), children were asked to perform expiration with a continuous pressure after a full inspiration into mouthpiece of spirometer, as well as a full inspiration after a full expiration. This process was repeated three times in order to reduce errors. Spirometry was performed 1.5 months after tonsillectomy. Spirometric parameters including FIF50%, FEF/ FIF50%, FEV1/ PEFR, and FEV1/ FEV0.5 measured before and after surgery were recorded along with data on age, gender, report of patients, and tonsil size. 

This study was approved by the Ethics Committee of Kerman's Medical Science University (Code Number K/91/268). No additional costs were incurred on patients as regards spirometry testing, and patients were informed that their lack of consent did not affect their typical treatment. All data were analyzed using SPSS Software (version 19) and central descriptive measures. Data were compared by performing a paired t-test; statistical tests were considered significant at the 0.05 level.

## Results

This study examined 50 patients suffering from enlarged palatine tonsils: 18 patients (36%) with +1 tonsil size, 18 patients (36%) with +2 tonsil sizes, seven patients (14%) with +3 tonsil size, and seven patients (14%) with +4 tonsil size. Thirty-three patients (66%) were female and 17 (34%) were male. The mean of age the patients was 9.7±2.97 years (range, 7–18 years). Moreover, 25 patients (50%), 17 patients (34%), and eight patients (16%), respectively, reported obstructive symptoms, recurrent tonsillitis, and both symptoms. Table 1 shows demographic characteristics of the patients enrolled in this study. Table 2 shows the results of spirometry with respect to FIF50%, FIF50%/FEF50%, FEV_1_/PEF and FEV_1_/FEV_0.5_ before and after surgery. 

**Table 1:** Demographic characteristics on the basis of tonsil size

**Tonsil size**	**Male/female**	**Age (years)**	**Obstructive**	**Recurrent tonsillitis**	**Both**
1+	7/11	10.72±3.25	5	10	3
2+	5/13	9.11±2.58	6	7	5
3+	2/5	9.28±3.14	7	0	0
4+	3/4	9.00±2.88	7	0	0

In patients with +1 tonsil size, FIF50% and FEF/FIF50 decreased after surgery. Conversely, FEV_1_/PEFR and FEV_1_/FEV_0.__5_ increased after surgery. In patients with +2 tonsil size, FIF50 increased after surgery. A similar increase was observed in FEV_1_/PEFR and FEV_1_/FEV_0.5_; however, mean FEF50%/FIF50% decreased. Patients with +3 tonsil size experienced an increase in FIF50% after surgery.

Conversely, FEV_1_/PEFR, FEV_1_/FEV_0.5_, and FEF 50%/ FIF50% decreased in all patients after surgery; although none of these differences were statistically significant. 

Conversely, as far as patients with +4 tonsil size were concerned, the increase in mean FIF50 and the decrease in FEV_1_/PEFR, FEV_1_/FEV_0.5_ were statistically significant and in favor of improved pulmonary performance after surgery (Table.2). 

**Table 2:** Results of spirometry on the basis of tonsil size

**Parameter**	**Tonsil size**			
	1**+**	2**+**	3**+**	4**+**
Pre FIF50%	2.31±1.63	2.31±1.63	2.31±1.63	1.74±0.85
Post FIF50%	1.95±1.11	2.35±1.10	1.88±0.77	2.90±1.26
P-value	0.087	0.177	0.75	0.001
Pre FEF50%/FIF50%	1.33±0.49	1.18±0.65	1.66±0.20	1.64±021
Post EF50%/FIF50%	1.25±0.42	1.15±0.48	1.27±0.46	0.66±0.34
P-value	0.519	0.833	0.380	0.001
Pre FEV_1_/PEFR	0.47±0.15	0.47±0.14	0.60±0.18	0.62±0.12
Post FEV_1_/PEFR	0.65±0.54	0.54±0.15	0.47±0.10	0.37±0.08
P-value	0.205	0.077	0.124	0.002
Pre FEV_1_/FEV 0.5	1.15±0.39	1.16±0.29	1.31±0.26	1.33±0.15
Post FEV_1_/FEV_0.5_	1.19±0.41	1.31±0.18	1.19±0.15	1.09±0.11
P-value	0.542	0.060	0.336	0.007

## Discussion

This study revealed an increased mean FIF50% and a decreased mean FEV_1_/ PEFR, FEV_1_/FEV_0.5_ and FEF50%/FIF50% after surgery in patients with +4 tonsil size. These statistically significant changes could improve the pulmonary performance of the patients involved. Accordingly, spirometry may be used as a simple test for diagnosing upper airway obstruction in patients displaying no clinical symptoms of adenotonsillar hypertrophy. 

Tonsillectomy is a common type of surgery in children. The frequency of this surgical technique varies from one region to another, and from one country to another (15-17); perhaps due to differences in training and education among general physicians, pediatricians, and otolaryngologists with respect to recurrent tonsillitis and conditions of upper airway obstruction (18).

Family characteristics and patient performance also affect the decision to perform tonsillectomy (19). Tonsillectomy is mostly used for the treatment of children aged 3–14 years, which is consistent with our findings. 

As concerns gender, adenoidectomy is performed in boys 1.5 times as frequently as in girls, and tonsillectomy is conducted in girls one-third more frequently than in boys (20). Our finding suggests that girls underwent tonsillectomy 1.9 times more frequently in comparison with boys. 

Obstructions and tonsil infections are the two main causes of adenoidectomy and tonsillectomy(21,22). Obstruction may occur in the airway route in the nasopharynx and oropharynx or in the swallowing route in the oropharynx. Chronic and recurrent infections may also affect the middle ear, mastoid air cells, nose, nasopharynx, adenoid, paranasal sinuses, oropharynx, tonsil, pretonsillar tissue, and lymph nodes of the neck. Loss of appetite, unexplained changes in weight, malignant tumors of the tonsils, or uncontrollable bleeding of the tonsils are other indications for tonsillectomy (23,24). In this study, all patients suffering from obstruction and recurrent tonsillitis underwent surgery. 

Upper airway obstruction caused by adenotonsillar hypertrophy tends to lead to sleep apnea, sleep disorders, pulmonary hypertension, mental retardation, dental anomalies, and speech disorders. As a result, patients often undergo tonsillectomy (25). Upper airway obstruction can be diagnosed by use of non-invasive techniques such as PFTs in cases where there is no clinical or radiological evidence of obstruction. Therefore, these tests can significantly impact upon decisions to perform tonsillectomy (13). 

One concern of otolaryngologists is the appropriate treatment of tonsil hypertrophy when symptoms of this disease are not recorded in the patient’s history and there are no explicit suggestions of them. One of the strategies in these cases is polysomnography, which is used for the diagnosis of obstructive sleep apnea and is regarded as an excellent diagnostic tool (26,27). Difficulties associated with this technique include lack of access to the required materials and laboratories in all regions, high costs, large time demands, the unwillingness of children to sleep in unfamiliar places, and their lack of comfort at the time of sleeping (26). 

PFTs are a useful tool for making decision relating to adenotonsillectomy. Studies demonstrate that in upper airway obstruction, air flow and lung volume decrease because of tonsillar hypertrophy. This decrease is observed to a greater extent in effort-based parameters. As the main difficulty associated with air flow obstruction created by adenotonsillar hypertrophy is related to the inspiration phase, this condition causes a reduction in FIF50%. This parameter is expected to increase after tonsillectomy, while the ratio of FEF50% FIF50% decreases (13). Reduction of expirational flow is a crucial diagnostic parameter, caused by a lower air current construction because of adenotonsillar hypertrophy. However, FEV_1_ is generally normal because it is dependent to a smaller extent upon respiratory inspirational function. Therefore, the ratio FEV_1_/PEFR is used, which is increased by obstruction and is expected to decrease after surgery. Also FEV_1_/FEV_0.5_ can be used as an indication of obstruction. During obstruction, FEV_0.5_ decreases to the greatest extent and consequently FEV_1_/FEV_0.5_ increases, which is expected to decrease after surgery. 

The greatest difficulty during airway obstruction arises in the inspiration phase, and therefore FIF50% is expected to increase after surgery to the greatest extent. Thus, in this study, in patients with tonsil size of +2, +3, and +4, FIF50% increased after surgery. In other words, we observed changes in this parameter due to an increase in obstruction. Ultimately, the mean of change in this parameter in the case of patients with a tonsil size of +4 was statistically significant after surgery. Our findings in patients with a tonsil size of +3 and +4 were consistent with the findings of Yadav et al. investigating changes in PFTs in patients suffering from obstruction. The difference between patients with +1 and +2 tonsils and patients with +3 and +4 tonsils may lie in the fact that some members of the former group displayed symptoms of pharyngitis rather than obstruction. Moreover, younger patients tended not to co-operate with the operator of the spirometer, which could also contribute to this difference. To identify patients for tonsillectomy, further studies are required in order to evaluate the impact of spirometry upon larger sample sizes and in patients with larger tonsils. 

## Conclusion

As our findings suggest, tonsillectomy can greatly relieve the symptoms of obstruction in patients with tonsils larger than +3. Additionally, spirometry can identify patients with +3 and +4 tonsils needing to undergo tonsillectomy. This tool is especially useful for patients who do not display clinical symptoms of obstruction, such as snoring and cessation of breathing at sleep. Accordingly, spirometry may be used as a simple test to diagnose upper airway obstruction. 
